# Response Efficacy and Action to Prepare for Disasters With Different Lead Time: Age Differences

**DOI:** 10.1093/geroni/igab046.1359

**Published:** 2021-12-17

**Authors:** Zhirui Chen, Zhen Cong

**Affiliations:** University of Texas at Arlington, Arlington, Texas, United States

## Abstract

This study investigated how disaster types, namely those with short and longer warning lead time, contextualized individuals’ preparatory action, especially as associated with their response efficacy and age. The working sample included 1,467 respondents from the 2017 U.S. National Household Survey. Logistic regressions showed that individuals with higher levels of response efficacy were more likely to prepare after learning information about how to prepare. Respondents in areas prone to short lead-time disasters were less likely to prepare than those in longer lead-time disasters areas. Response efficacy was more important for action taking for short lead-time disasters, which was observed only among older adults when older and younger adults were examined separately. These findings revealed the impacts of disaster types and response efficacy on disaster preparedness and older adults’ unique vulnerability and resilience, which could guide policymaking and interventions to promote national disaster preparedness tailored to regional peculiarities.

